# Lower respiratory rate during sleep in children with Angelman syndrome compared to age-matched controls

**DOI:** 10.1186/s13023-025-03553-2

**Published:** 2025-04-08

**Authors:** Leo Gschwind, Sebastian Camillo Holst, David Nobbs, Florian Lipsmeier, Katalin Buzasi, Ponghatai Boonsimma, Alexander Rotenberg, Vitaliy Kolodyazhniy, Jörg Felix Hipp

**Affiliations:** 1 Roche Pharma Research and Early Development, Data and Analytics, Roche Innovation Center Basel, F. Hoffmann–La Roche Ltd., Basel, Switzerland; 2 Roche Pharma Research and Early Development, Neuroscience and Rare Diseases, Roche Innovation Center Basel, F. Hoffmann–La Roche Ltd., Basel, Switzerland; 3 Roche Informatics Solutions, Data, Analytics and Research, F. Hoffmann–La Roche Ltd., Basel, Switzerland; 4 Fortrea Development Limited, Budapest, Hungary; 5 Department of Neurology, Boston Children’s Hospital, Harvard Medical School, Boston, MA USA

**Keywords:** Angelman syndrome, Sleep disorders, Digital biomarkers, Sleep mat, Respiration, Respiratory rate

## Abstract

**Background:**

Angelman syndrome (AS) is a rare genetic neurodevelopmental disorder caused by the absence of a functional UBE3A gene, leading to developmental, behavioral, and medical challenges. Sleep disturbances, including sleep-disordered breathing, are common in AS. This study, for the first time, investigates nocturnal respiration in individuals with AS and healthy controls at home in a long term setting.

**Methods:**

A non-invasive ballistocardiography-based (BCG) sleep monitoring device (“sleep mat”) placed under the participants’ mattresses, was used to remotely monitor children with AS aged 1 to 12 years (6.0 ± 3.2 years, *n* = 40) and age-matched typically developing controls (TDC) (6.2 ± 3.5 years, *n* = 20) for approximately 12 months. The sleep mat recorded physiological signals during times in bed. We applied fast-Fourier transformation (FFT) to exclude segments without a clear respiratory signal, thereby minimizing the impact of large body movements, wakefulness, or seizure activity. Moreover, polysomnography (PSG) was collected for up to three nights for each participant in their home. Clinical characteristics, genotype, and Bayley Scales of Infant and Toddler Development^®^ (Bayley-III) were also analyzed.

**Results:**

The average median BCG-derived respiratory rate over the entire study duration was significantly lower in AS compared to TDCs (Cohen’s d = 1.31). PSG-derived respiration data corroborated the lower breathing rate in AS (Cohen’s d = 0.77) and revealed a strong correlation between BCG and PSG derived respiration (*r* = 0.85) and thus a strong convergent validity of the sleep mat against “gold standard” measures. Next, we defined two groups of AS individuals based on their respiratory rates: a normal respiration group with rates above the minimum in TDC, and a low respiratory rate group with rates below the TDC group’s minimum. A higher prevalence of respiratory abnormalities was observed in deletion carriers (55.2%) versus non-deletion carriers (9.1%). Pulse oximetry data indicated lower oxygen saturation levels in AS individuals (Cohen’s d = 1.60). Moreover, lower Bayley-III scores were observed in the low respiration group, suggesting a link between respiratory dysfunction and neurodevelopmental outcomes in AS. Medication use, particularly antiepileptic drugs, was found to suppress respiratory rates, highlighting the complex interplay between concomitant medication use, genotype, and sleep in AS.

**Conclusion:**

Our study provides the first long-term observational evidence of a persistent bradypnea-like phenotype in individuals with AS, which may have significant implications for their clinical management. The successful use of the sleep mat device as a non-invasive physiological ambulatory monitoring tool demonstrates its potential as a digital health technology for detecting respiratory abnormalities in pediatric neurodevelopmental disorders. These findings should be further assessed and may have biomarker and clinical utility in AS, particularly in relation to seizure management and cognitive development.

**Supplementary Information:**

The online version contains supplementary material available at 10.1186/s13023-025-03553-2.

## Background

Angelman syndrome (AS) is a genetic neurodevelopmental disorder with a prevalence of approximately 1 in 20,000 births [1–3]. AS is caused by the loss of function from the maternally inherited allele of the gene encoding ubiquitin-protein ligase E3A (UBE3A) located on chromosome 15q11.2-q13.1. In neurons, UBE3A is imprinted, i.e. expressed only from the maternal allele and plays an important role in neuronal development and brain function, with maternal deletions including the UBE3A gene (~ 70%) being most common [4]. Clinical characteristics of AS include global developmental delay with absence or near absence of speech, movement and balance problems, unique behavior with outbursts of laughter and hyperactivity, epileptic seizures and sleep disorders [5, 6]. Sleep problems, which are part of the diagnosis criteria for AS, are a major stressor for caregivers [7, 8]. These sleep disturbances include difficulty falling asleep, frequent nighttime awakenings, and reduced overall sleep duration [9], with comorbidity rates of up to 80% [10]. Moreover, REM sleep is significantly reduced in AS and can even appear absent, further highlighting the severity of sleep disturbances in this population [11, 12]. Like in many other neurodevelopmental disorders [13], sleep disordered breathing (SDB) is considered a contributing factor to sleep problems in AS [14–16]. SDB encompasses a range of respiratory issues, including obstructive sleep apnea (OSA) and central sleep apnea, which can lead to frequent arousals and fragmented sleep [17]. In AS, both sleep disordered breathing and frequent apnea events have previously been reported [11], and respiratory system impairments are often listed as one of the main causes for hospitalization of patients with AS [18]. There have also been isolated case reports of abnormally slow breathing rates in AS, referred to as bradypnea [16, 19].

Nevertheless, objective evidence for SDB in AS is limited, as standard measurement modalities such as respiration belts, air-/thermo-sensors and oximetry are often poorly tolerated in this population, especially during long term monitoring. This is largely due to the discomfort caused by wearable sensors, which can be problematic for individuals with AS who often have elevated sensory sensitivity and are generally difficult to instruct [20, 21]. Consequently, there is a significant gap in our understanding of sleep behavior including long-term respiratory patterns during sleep in individuals with AS. Improved understanding of these features may inform clinical care and treatment monitoring and could be considered for efficacy analyses in clinical trials. To address these challenges, we used a highly sensitive ballistocardiography-based sleep monitoring system referred to as a “sleep mat”, which was placed beneath the participants’ mattress. Besides heart rate monitoring, this system enabled us to successfully measure respiration rates in young children with AS over extended periods of sleep and time in bed, imposing minimal burden to patients [22]. After initial training by the study personnel, the caregivers implemented the sleep mat in the home setting. Besides low burden compared to conventional assessments of respiration, this approach allows assessing participants in their regular environment and usual sleeping conditions across long durations. Additionally, polysomnography (PSG) with a limited set of sensors (including respiration belts) was recorded during single night home visits, which allowed us to validate respiration data from the sleep mat against this gold standard.

The data and results presented in this paper have been collected in the context of the prospective, observational, yearlong study FREESIAS as part of the feasibility assessment of digital health technologies [23]. The aim of these analyses were to assess respiration patterns of AS individuals during sleep, to evaluate breathing disturbances, the feasibility of long term data collection in an AS population, and potential differences between AS patients and healthy controls. To our knowledge this is the first long term study investigating respiration during sleep in a pediatric AS population with at home monitoring.

## Methods

### Study design and participants

This manuscript analyzes data from individuals with AS and control participants acquired within the FREESIAS observational study (BP40654), a longitudinal investigation aimed at assessing the feasibility and utility of in-clinic and at-home measures of key AS symptoms [23]. The study protocol was prospectively designed to be compatible with the Angelman Syndrome Natural History Study (AS-NHS; NCT04507997). Several clinical outcome assessments and digital health technologies were utilized in the FREESIAS study, including a sleep mat based ballistocardiography (BCG) (Emfit Ltd, Vaajakoski, Finland) and overnight PSG with a limited set of sensors to reduce burden to participants that included electrocardiogram (ECG), respiration belts, 10/20 EEG and pulse oximetry sensors. PSG assessments were planned at baseline (following Clinic Visit 1), 12 months later (before Clinic Visit 2), and during one intermittent home visit around 2 months. In addition, a sponsor-provided smartphone enabled caregivers of participants with AS to register all seizures that occurred during the study in a trial-specific seizure diary (see [23] for further details). Finally, at home, continuous, and holistic sleep data were collected through the smartphone-based sleep diaries and BCG-based sleep mat. 55 individuals with AS (*n* = 40 children age 1–12 years, median: 6 years; *n* = 15 adults age 18–38 years, median: 24 years) and *n* = 20 typically developing controls (TDC) individuals (children 1–12 years, median: 6 years) were enrolled across six USA sites from September 2019 to May 2021 (Table 1; see also: [23]). Here, we focus on pediatric participants, the subpopulation with AS and TDC, resulting in 20 TDC siblings and 40 children with AS. The AS population comprised 14 (35%) deletion class 1, 13 (32.5%) deletion class 2, 2 (5%) with atypical deletions, 4 (10%) with a *UBE3A* mutation, 5 (12.5%) with uniparental paternal disomy and 2 (5%) with imprinting defects. The age range of analyzed participants was 1 to 12 years (see Table 1 for details). Our analysis focused on respiratory parameters collected via the sleep mat and PSG, as well as clinical characteristics, genotypes and Bayley Scales of Infant and Toddler Development^®^– Third Edition (Bayley-III).

### Overnight at home digital ballistocardiography (sleep mat) assessments

To objectively assess sleep as well as physiological and activity parameters on a daily basis in participants’ home environment, we utilized a digital sleep mat (Emfit Ltd, Vaajakoski, Finland) based on BCG. The mat was placed under participants’ mattresses for the entire 12-month duration of the study [22]. “Adherence”, measured as the percentage of days per week the mat was used throughout the approximately 52 week study period, was found to be 59% for individuals with AS and 53% for typically developing controls (see [23]). The digital sleep mat provides a unique unobtrusive solution to collect long term data in a home setting. Once the sleep mat sensor is placed under the mattress and plugged into a power outlet, its integrated 3G module automatically transmits collected data securely to the vendor’s server. The BCG sensor is able to register minute body movements, which it uses to extract respiratory and heart rate information in 4 s bins and as an average across each recording period [22]. By focusing our analysis exclusively on high-quality data segments as pre-defined by the Emfit Ltd device [22], we excluded data segments without a clear sinusoidal respiratory signal and thereby reduced data with large body movement or clonic seizure activity. Moreover, to further improve the integrity of the respiratory rate data (see Supplementary Methods 1 for full details) sleep mat recordings underwent additional quality control where we eliminated any 4 s bins with a respiration rate below 8 breaths per minute and above 25.5 breaths per minute. The lower threshold was set to account for potential poor data quality, signal loss, or the presence of apnea events, whereas the upper threshold was defined to match the upper limit of the device (supplementary methods 1). Signal loss during respiratory monitoring can occur due to excessive participant movement or if the participant, particularly a child, moves out of the sleep mat sensor’s coverage area. While this selective approach enhances the accuracy of our respiratory rate measurements, it inherently precludes the quantification of apnea events and further reduces the probability of collecting data during clonic seizure events. In total we were able to collect about 84’409 h of BCG respiratory rate data of which roughly 52’497 h (~ 62%) across 7’856 unique nights for 60 individuals (AS = 40, TDC = 20), remained available for analysis after QC and age matching (see also supplementary methods 1) This added up to 867 ± 529 h (median: 854 h; range 22–2334 h) of data for each individual with AS and 891 ± 675 h (median: 725 h; range 120–2653 h) for each TDC.

### Home visits PSG

During the 12-month study, 3 home visits for each study participant were planned. Due to Covid-19, only 90 of the planned visits took place. Each visit included supervised PSG data acquisition at home with a specialist present, who visited the patients’ home and was responsible for ensuring accurate collection of data. Generally PSG with a multitude of wired sensors attached to the body, is a challenge for pediatrics and even more so for pediatrics with intellectual disability. Furthermore, individuals with AS often have elevated sensory sensitivities [24] and therefore poorly tolerate assessments such as PSG. As a result, it is crucial to implement stringent quality control measures (see Supplementary Methods 2). Before implementing PSG quality control measures, respiratory data from 76 unique nights (approximately 679 h of data) were available. After the quality control process, we were able to retain around 493 h, which accounts for approximately 73% of the original dataset across 65 unique nights, which was deemed suitable for further analysis. Additionally, we successfully collected 23 unique nights (approximately 245 h) of blood oximetry (SpO2) data using a finger sensor in compliant participants. Applying quality control measures to this dataset resulted in approximately 174 h available data across 19 unique nights for analyses, which represents roughly 71% of the data. After the exclusion of adult AS individuals the PSG respiration dataset comprised 56 nights (AS *N* = 24, TDC *N* = 12) and for PSG SpO2 19 nights (AS *N* = 9, TDC *N* = 7). For more information see also Supplementary Methods 2.

### Clinical visits & cognitive assessments

At the beginning and end of the study, on site clinical visits were planned to assess participants. Whereas all 75 study participants completed clinic visit 1, 71 individuals completed clinic visit 2. Detailed information can be found in the main study publication [23].

The Bayley Scales of Infant Development (BSID; BAYLEY-III) was used to assess cognition, communication and motor skills in individuals with AS [25, 26]. Here we used scores from the 5 BSID domains as a measure of symptom severity for investigating the relationship with the identified respiratory phenotype. To this end scores were age corrected as previously described [25]. In brief, by modeling random intercepts per participant and per study site, and by using a third-order mean-centered orthogonal poly-logarithmic function of log-age as fixed effect, we capture the nonlinear developmental trajectories observed on the Bayley-III. Fits were performed separately for Deletion and Non-deletion AS. Predictions based on the model were then removed from observed data. The thus age-corrected values can be interpreted as a severity score relative to an individual with AS with average performance.

### Concomitant medication use

Upon enrollment and at each subsequent site visit, a detailed account of each participant’s use of non-investigational drug products and non-pharmacologic interventions was obtained. This was accomplished through interviews with caregivers and by reviewing patient medical records. Any changes in medication use or the occurrence of adverse events were promptly recorded in the electronic Case Report Form (eCRF), which provided a secure, standardized repository for relevant participant data. To explore association with the use of concomitant medications and nocturnal respiration, we systematically categorized the daily medications used by AS individuals based on their pharmacological properties and potential impact on respiratory rate during sleep. The medications were divided into three groups. (1) Benzodiazepines (benzos): This group included clobazam and clonazepam, which are known for their sedative properties with the capability to depress respiration, particularly in populations at risk for respiratory dysfunction [27–29]. (2) Psychotropic (CNS relevant) drugs: This broad category encompassed a range of medications with primary effects on the CNS, including amitriptyline, aripiprazole, buspirone, cannabidiol, clonidine, diphenhydramine, ethosuximide, gabapentin, guanfacine, lamotrigine, levetiracetam, melatonin, trazodone, risperidone, valproate, zonisamide. These drugs were selected based on their lack of reported impact on sleep respiration rate, despite their CNS activity. (3) Others: This group consisted of medications presumed to have no direct effect on the respiratory system, such as cetirizine, cyproheptadine, esomeprazole, famotidine, glycopyrrolate, lansoprazole, ranitidine, sertraline.

For each AS patient, medication usage was recorded as a binary variable, indicating regular consumption (at least once per day) of any drug within the aforementioned categories. This approach facilitated the creation of a binary matrix representing the presence or absence of each drug category for each participant. Subsequently, we grouped the data into a secondary matrix to compare the frequency of each drug category between the low and normal respiratory rate groups among individuals with AS. This methodology allowed for the quantification of medication usage patterns and the evaluation of potential associations between drug consumption and respiratory rate during sleep in the AS population.

### Statistical analysis

The combination of small unequal sample size, unequal variance, non normal distributed data and nonlinear age effects lead to the decision to use nonparametric Brunner-Munzel tests [30, 31] (referred to as BMT; see also the supplementary material) for group comparisons (AS vs. TDC). For comparisons of different modalities, nonparametric Spearman rank correlation coefficients were used, with parametric Pearson correlation coefficients also reported to elucidate consistency of our findings. For contingency tables we used the conservative fisher’s exact test.

## Results

### Impaired nocturnal respiration in individuals with AS

We investigated nocturnal respiration in individuals with AS using both at-home digital sleep mat technology and in-lab polysomnography (PSG). The analysis included data from 40 individuals with AS and 20 TDCs, with no significant differences in age, gender, height, weight, or body mass index between the groups (Table 1; Supplementary Results Figs. 2 and 3). The AS cohort comprised approx. 70% with deletion genotype (15q11.2-q13 deletion) and approximately 30% with nondeletion genotypes (*UBE3A* mutations, UPD, ICD, see methods). The majority (70%) of AS participants were diagnosed with epilepsy, and a small fraction of patients and controls had a history of sleep-disordered breathing (Table 1).

**Table 1:** Clinical characteristics of the study population

	All AS (*N* = 40)	AS deletion (*N* = 29)	AS nondeletion (*N* = 11)	TDC (*N* = 20)
Age (years)	6.0 ± 3.2	5.7 ± 3.3	6.8 ± 2.9	6.2 ± 3.5
Gender [Male (female)]	24 (16)	17 (12)	7 (4)	10 (10)
Height (cm)	114.4 ± 19.2	111.9 ± 19.4	120.9 ± 17.8	118.5 ± 24.9
Weight (kg)	23.3 ± 9.9	22.0 ± 10.5	26.7 ± 7.6	23.9 ± 10.5
BMI (kg/m^2^)	16.9 ± 2.3	16.6 ± 2.5	17.9 ± 1.6	16.5 ± 1.93
Diagnosis of epilepsy	28/40 (70%)	23/29 (79%)	5/11 (45%)	-
Previous diagnosis of sleep disordered breathing	2/40 (5%)^a^	1/29 (3%)	1/11 (9%)	1/20 (5%)^b^
Benzodiazepine use daily	19/40 (47.5%)	18/29 (62.1%)	1/11 (9%)	-
Other psychotropic drug (s) use daily	30/40 (75%)	21/29 (72.4%)	9/11 (81.8%)	-

Demographic characteristics of available participants across individuals with Angelman syndrome (AS) and typical developing controls (TDC). Data presented as mean ± standard deviation. ^a^Two AS participants were diagnosed with obstructive sleep apnea. ^b^One typically developing participant was diagnosed with sleep apnea (unspecified). Statistical analysis performed using Brunner Munzel tests or fisher exact tests where appropriate which revealed no significant differences between patients and TDCs

**Fig. 1:**
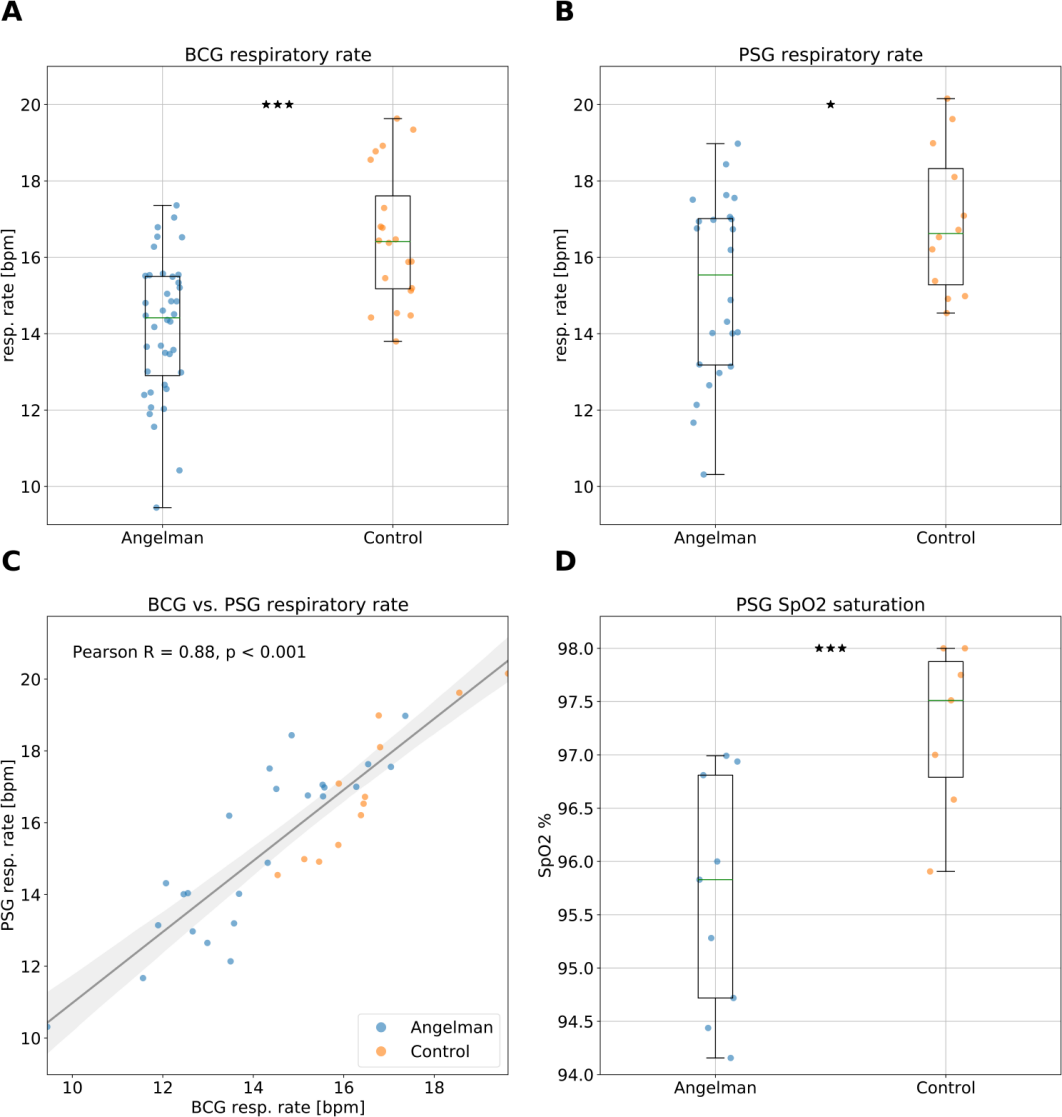
Impaired nocturnal respiration in individuals with AS. Nocturnal respiration patterns in AS (blue dots, 5,589 total nights) and typical developing controls (TDC) (orange dots, 2,267 total nights). **A**. Sleep mat ballistocardiography (BCG) detected nocturnal respiration rate (RR) for each participant across the entire study duration. **B**. Polysomnography (PSG) detected RR in a subset of participants (where available), confirming a pattern of lower RR in AS. **C**. Correlation between RR measurements from the sleep mat BCG and PSG data, validating the sleep mat’s reliability. Spearman rho = 0.85, *p* < 0.001; Pearson *r* = 0.88, *p* < 0.001 **D**. Reduction in oxygen saturation (SpO2) levels in AS individuals compared to TDC. (*: *p* < 0.05, **: *p* < 0.01, ***: *p* < 0.001)

Using the sleep mat, we observed a significantly lower nocturnal respiration rate (RR) in the AS group compared to TDCs over 5,589 nights, with a median RR of 14.4 ± 1.8 bpm (breaths per minute) for AS and 16.4 ± 1.8 bpm for TDCs (Fig. 1A). This difference was statistically significant (*p* < 0.00001) with a large effect size (Cohen’s d = 1.31). A sensitivity analysis comparing the six individuals with most frequent seizure reports from the seizure diary (176 / 186 seizure events, RR: 14.26 ± 1.76), with the remaining AS group (*n* = 34, RR: 14.13 ± 1.85; *p* = 0.97), confirmed the robustness of this finding and detailed that even when excluding ~ 94.6% of total seizure reports, the AS group still shows a significantly lower RR compared to TDC individuals (*p* < 0.00001).

Respiration data was also available from a breathing belt as part of PSG recordings that are available for a subset of participants. Respiration belts can be considered a gold standard for measuring respiration. We therefore investigated the agreement between the sleep mat and the breathing belt. We found a strong correlation between the RR derived from the sleep mat and the PSG, indicating high consistency between the two modalities (Spearman rho = 0.85, *p* < 0.001; Pearson *r* = 0.88, *p* < 0.001; derived from 56 nights with at least 3 h of respiration belt data and the RR rate from the sleep mat across the entire study; Fig. 1C). Furthermore, the limited respiration belt data confirmed the decreased RR for AS compared to TDCs, although the differences were slightly less pronounced. The AS group (average median RR = 15.5 ± 2.4 bpm) showed a lower RR compared to TDCs (average median RR = 16.6 ± 1.9 bpm) over 35 nights (Fig. 1B; *p* < 0.05, Cohen’s d = 0.77).

### Reduced RR is paralleled by reduced blood oxygen saturation

We next investigated if the lower RR had an impact on the blood oxygen levels. For *n* = 16 subjects, SpO2 levels were acquired during the PSG assessments. We found a significant reduction in nocturnal oxygen saturation (SpO2) in the AS group (median SpO2 = 95.8%) compared to TDCs (median SpO2 = 97.5%) (Fig. 1D; *p* < 0.001, Cohen’s d = 1.60). In line with these group differences, we found a significant correlation between RR and SpO2 in the subset of patients where both measures were available (*n* = 16, Pearson *r* = 0.62, *p* < 0.011; Spearman rho = 0.53, *p* = 0.034). In sum, our results demonstrate a clear reduction in nocturnal RR, going in hand with reduced SpO2 in individuals with AS, which may have implications for their overall health and quality of life.

### The AS RR phenotype is most pronounced in AS individuals with deletion genotype

We next explored the potential genetic subtype dependence (deletion vs. nondeletion AS) of the RR phenotype. To this end, we split the AS group into two subgroups based on their RR. The normal RR group (normRR), included AS individuals with RR within the range observed in TDCs (RR > 13.8 breaths/min; *n* = 23/40, 57.5%), while the low RR group (lowRR), comprised those with respiratory rates lower than the minimum observed in TDCs (RR < 13.8 breaths/min; *n* = 17/40, 42.5%). The lowRR group was predominantly composed of individuals with deletion AS (16/17, 94%), resulting in a significant overrepresentation of the deletion AS genotype (Fisher’s exact test, *p* < 0.02; Fig. 3A).

### Confirmation of AS RR phenotype with external RR norm data

**Fig. 2:**
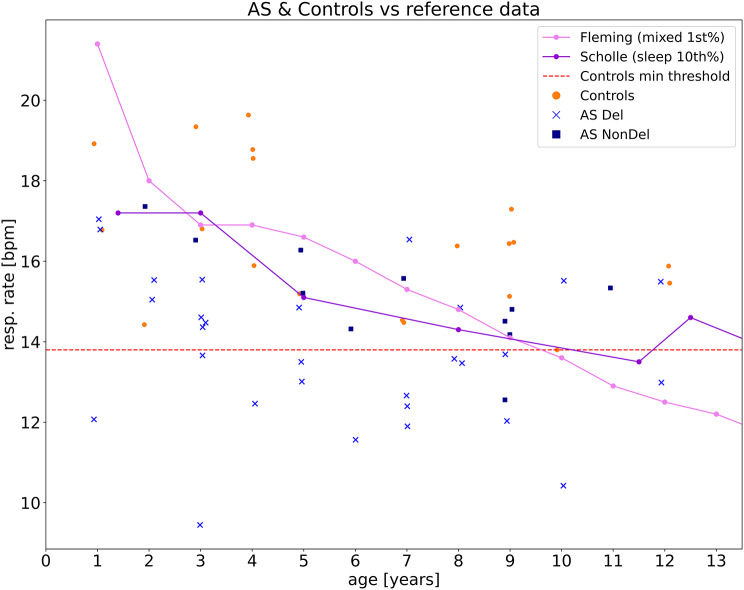
Respiration rates as a function of age. Respiration rates of AS individuals with deletions (Del: light blue crosses) and non-deletion genotypes (NonDel: dark blue squares), compared to age-matched controls (orange points) as a function of age. The red dotted line indicates the lowest respiration rate observed among controls in our sample at 13.8 breaths per minute (bpm). The pink line represents the 1st percentile normative respiration rate from Fleming et al., 2011, which includes data from children in mixed states (asleep and awake), while the purple line from Scholle et al., 2011, represents the 10th percentile for children during sleep. These lines serve as benchmarks for the lower bounds of normal pediatric respiration rates. Deletion carriers show a higher incidence of low respiration rates (Del: 23/29, 79.3% below Fleming et al.‘s 1st percentile; 24/29, 82.8% below Scholle et al.‘s 10th percentile) compared to non-deletion carriers (NonDel: 4/11, 36.4% and 2/11, 18.2% respectively; Fisher’s exact test: *p* < 0.03 for Fleming et al., *p* < 0.0003 for Scholle et al.). AS patients also have lower respiration rates than controls (AS: 27/40, 67.5% and 26/40, 65.0% below the respective percentiles; Controls: 7/20, 35.0% and 2/20, 10.0%; Fisher’s exact test: *p* < 0.03 for Fleming et al., *p* < 0.0001 for Scholle et al.). This suggests an increased risk of bradypnea AS individuals compared to the controls

The typical developing control population from within the study is well matched in terms of age and setup, however, the control group is relatively small. We therefore performed a complementary analysis utilizing external RR age-dependent normative data to further investigate the AS RR phenotype (Fig. 2). The normative data on low RRs was extracted from Fleming et al. [32], which provides the 1st percentile for children with mixed states (asleep and awake), and Scholle et al. [33], which offers the 10th percentile for children specifically during sleep. We found a high number of participants with deletion genotype that showed respiration rates below the age-corrected 1st percentile of mixed states from Fleming et al. (23 out of 29, 79.3%) and below the 10th percentile of sleep states from Scholle et al. (24 out of 29, 82.8%). In contrast, AS individuals with nondeletion genotypes were less likely to fall below these thresholds, with only 4 out of 11 (36.4%) below the 1st percentile and 2 out of 11 (18.2%) below the 10th percentile (AS deletion vs. nondeletion: *p* < 0.03 for Fleming et al., *p* < 0.0003 for Scholle et al.; Fisher’s exact test). This was in stark contrast to the control group, where only 7 out of 20 (35.0%) and 2 out of 20 (10.0%) fell below the respective percentiles (AS deletion vs. controls: *p* < 0.03 for Fleming et al., *p* < 0.0001 for Scholle et al., Fig. 2; Fisher’s exact test). These results, based on external norm data, are in line with our observations based on the within study controls and further confirm our observations of an AS RR phenotype that is most pronounced for deletion AS.

### Association of AS RR phenotype with drugs

**Fig. 3:**
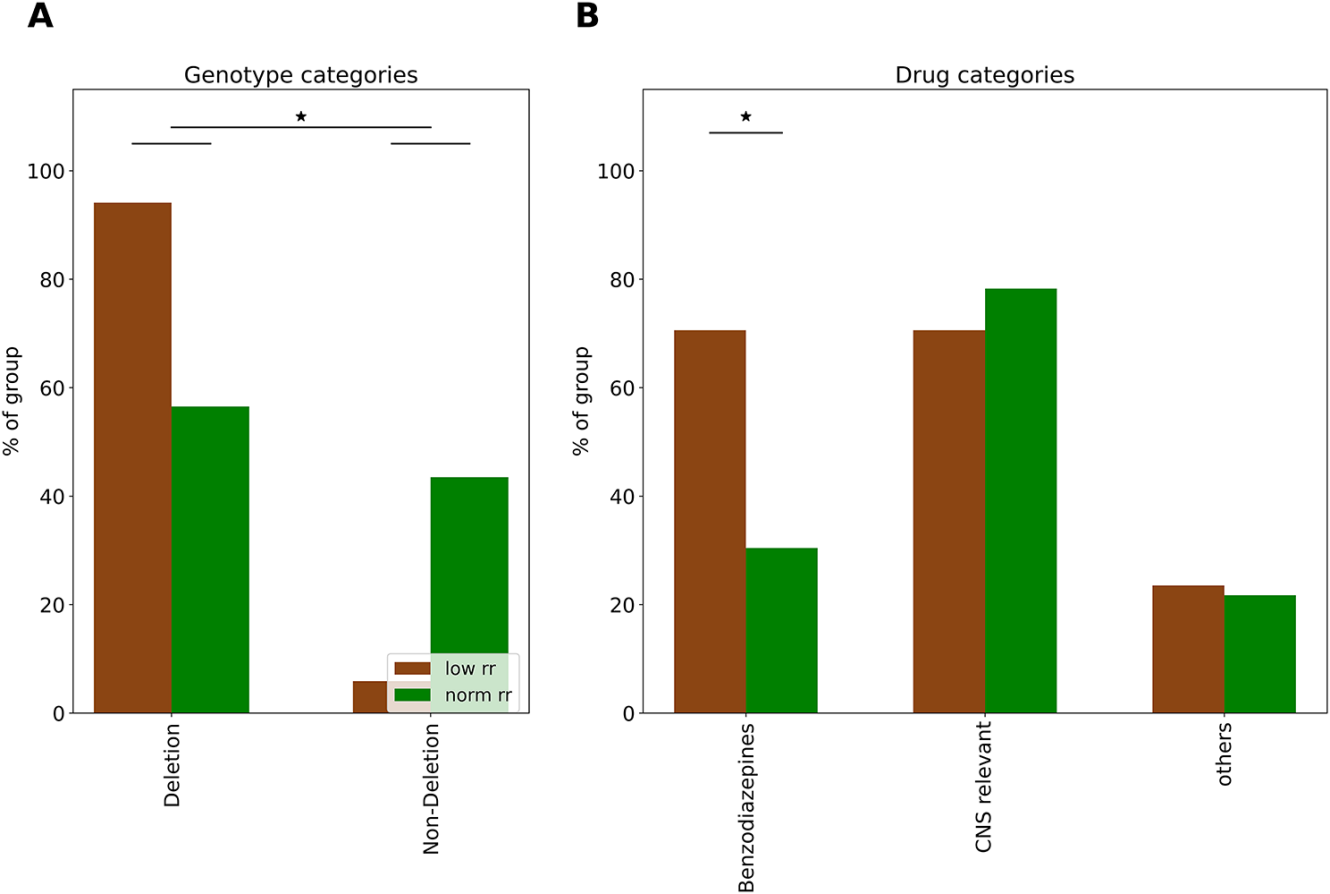
Distribution of genetic subtypes and medication usage in AS individuals with low or normal respiratory rates. Distribution of Genetic Subtypes and Medication Usage in AS Individuals with Low (lowRR: < 13.8 breaths/min) or Normal Respiratory Rates (normRR: > 13.8 breaths/min). (**A**) The distribution of genetic subtypes among AS individuals categorized as deletion (normRR: *n* = 13, lowRR: *n* = 16) and non-deletion (normRR: *n* = 10, lowRR: *n* = 1) carriers demonstrating a significant overrepresentation of deletion genotype carriers in the lowRR group (Fisher’s exact test, *p* < 0.02). (**B**) Stratification of medication usage among AS individuals in the normRR and lowRR groups. Medications are categorized into three groups: benzodiazepines (“benzos”), central nervous system (CNS) active drugs, and other medications with no primary respiratory action (see methods for details). The data details a significantly higher prevalence of benzodiazepine prescriptions in the lowRR group (Fisher’s exact test, *p* < 0.03), suggesting a potential association between benzodiazepine use and lower respiratory rates in AS individuals.(*: *p* < 0.05)

To assess the impact of concomitant medication (see also Supplementary Results Table 1) on RR in individuals with AS, we grouped medications into three distinct categories based on known effects on respiration and their primary pharmacological action (see methods). The first category, “Benzodiazepines” included benzodiazepines, which have been documented to suppress respiration in susceptible populations [27–29]. The second category, “CNS,” was designated for medications that, while not reported to affect sleep or respiratory rate, exert effects on the central nervous system. The third category, “others,” comprises medications that are not expected to have a direct impact on the respiratory system even though some may exert effects on the CNS (see methods). This classification allowed for a focused analysis of the potential influence of these drug groups on the respiratory patterns of AS patients during sleep. We created a binary matrix of all drugs per participant and grouped them into a second matrix to calculate the percentage per drug-group per respiratory-group (Fig. 3B). While no individual medication demonstrated significant differences in usage between groups (Supplementary Results Fig. 3), the aggregate administration of benzodiazepines was notably more prevalent in the low respiratory rate (lowRR) group (Fisher’s exact test, *p* < 0.03; Fig. 3B).

### Association between RR and clinical severity in AS

**Fig. 4:**
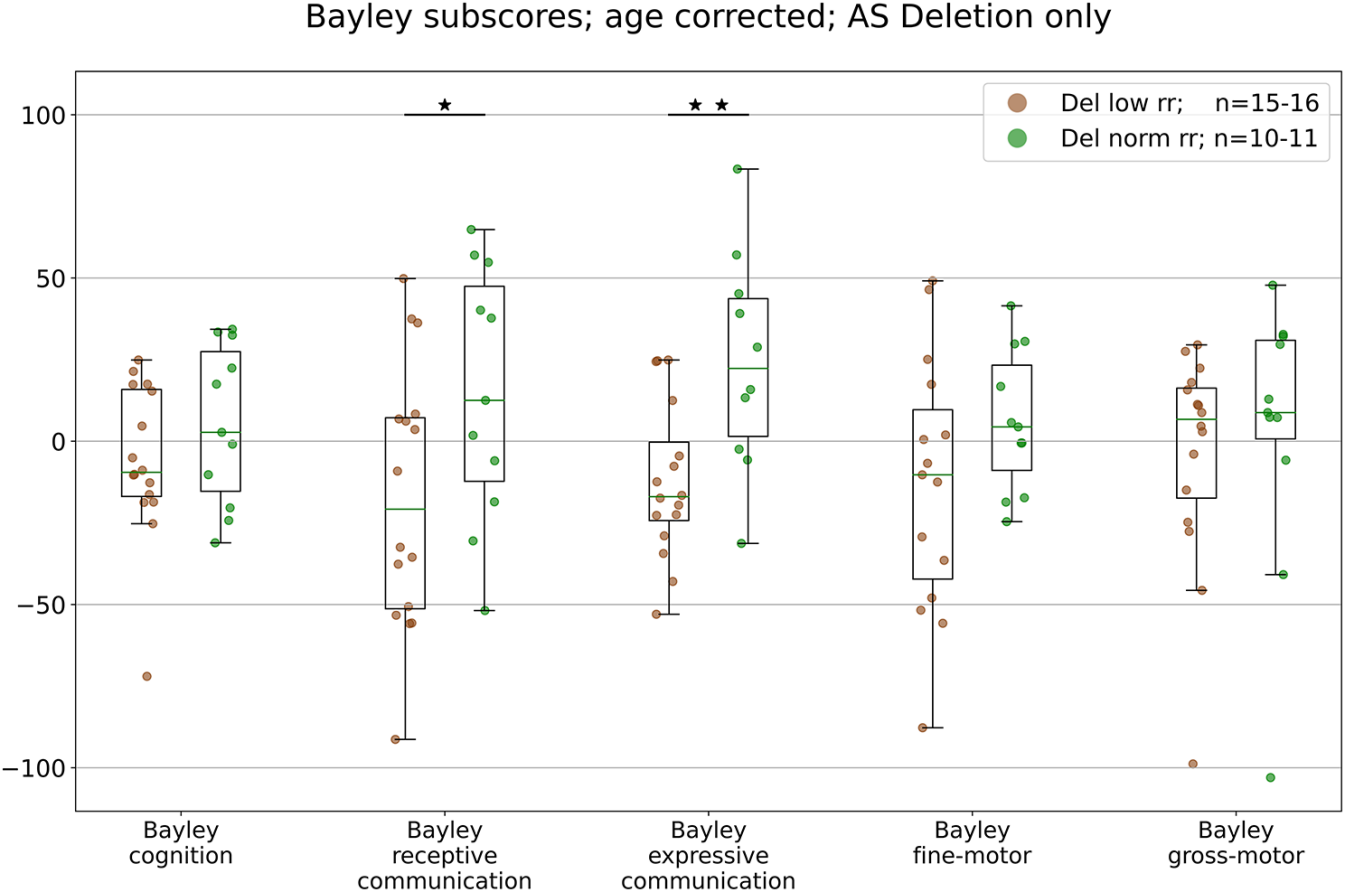
Cognitive outcomes on BSID in AS individuals by respiratory rate category. Age-corrected BSID growth score values for low RR and normal RR deletion AS subgroupsStars represent significant effects as detected by non-parametric Brunner Munzel testing (*: *p* < 0.05, **: *p* < 0.01)

In our final analysis, we examined the relationship between respiratory rates and symptom severity as assessed by the Bayley Scales of Infant and Toddler Development [34]. Specifically, we compared the five age-normalized Bayley scores (see methods) and the average across all scores between AS individuals in the low respiratory rate (lowRR) group and those in the normal respiratory rate (normRR) group. Given the AS RR phenotype is most pronounced in deletion AS and given low number of nondeletion AS for analysis, we restricted this analysis to deletion AS.Across all 5 scales, we found lower scores for the lowRR subgroup (Fig. 4), which reached significance for receptive communication (BMT = 2.3, *p* < 0.04) and expressive communication (BMT = 3.5, *p* < 0.004). These results suggest that lower respiratory rates in AS individuals with deletion genotypes may be associated with more pronounced deficits in communication skills.

## Discussion

We investigated nocturnal RR in individuals with AS, a population known to experience significant sleep disordered breathing and associated health complications [11, 14, 35–37]. Respiratory challenges in AS impact the quality of sleep and present a major cause of hospitalization [18]. Prior research in AS primarily focused on apnea events or relied on questionnaire-based assessments of sleep disturbances [11, 14, 35]. There have also been isolated case reports of abnormally slow breathing rates in AS, known as bradypnea [16, 19], but such a phenotype has never been objectively investigated in a larger, long term AS study. Here, we utilized longitudinal BCG-based assessments of RRs complemented by gold-standard breathing belt and pulse oximetry data to investigate nocturnal RRs. Our results revealed a nocturnal bradypnea phenotype in AS individuals when compared to typically developing age-matched controls that was most pronounced in deletion AS and was accompanied by reduced blood oxygenation as detected by pulse oximetry in a subset of individuals. These findings suggest a potential vulnerability to hypoxemia associated with the bradypnea phenotype, which could have implications for health and development in AS and may be of clinical relevance. Indeed, our most recent paper details enhanced T-wave alternance in AS compared to TDCs, which further supports the notion that AS individuals may have underlying cardiac abnormalities [38] that could be linked to the reported respiratory dysfunctions.

We used longitudinal BCG data from a “sleep mat” to measure RR. In a subset of participants, a respiration belt was used during polysomnography assessments and allowed comparison of the sleep mat derived RR data to what can be considered the gold standard. The high correlation between BCG and PSG derived RRs not only reinforces the accuracy of our observational approach but also confirms the validity of the sleep mat derived data. Furthermore, the sleep mat data is a remote assessment in the home setting with little participant burden. In sum, the BCG data from a “sleep mat” provides an accurate, low-burden, long-term monitoring tool for RR that is particularly valuable to study vulnerable populations like AS.

Our analysis revealed the most pronounced respiratory challenges in deletion AS. It is well established that AS individuals with the deletion phenotype have a more severe clinical manifestation [25, 39, 40]. As for other symptoms, this suggests that the additional genes affected by deletion AS, may play a critical role in the respiratory abnormalities in AS. The deletions typically encompass the 15q11.2-q13 region, which contains several genes beyond UBE3A, including a gene cluster of gamma-aminobutyric acid type A receptor (GABAA) subunits, including β3-, α5-, and γ3 (i.e., GABRB3, GABRA5, and GABRG3). GABAergic mechanisms are integral to inhibitory synaptic transmission in the central nervous system, and known to be involved in the control of respiratory rhythms [41, 42]. Reduced expression of these gene products may have a direct impact on respiratory depression and contribute to the more severe phenotype observed in deletion carriers. However, the bradypnea phenotype may also be a down-stream consequence of the more severe deletion AS phenotype (see section below on origin of bradypnea phenotype for further discussion).

Bradypnea, commonly defined in adults as fewer than twelve breaths per minute (bpm) [43], is generally higher in children who have a more rapid respiration [32]. Bradypnea is observed in various neurological clinical conditions, following the use of sedative hypnotics, and has been linked to sleep-induced seizures and the risk of sudden unexpected death in epilepsy (SUDEP) [44]. While specific reports of a bradypnea-like phenotype in other neurodevelopment disorders are rare, SDB is commonly reported across ASD [14, 45], Rett syndrome [11, 46], Fragile X [47, 48] and Prader-Willi syndrome [48, 49]. Our data is therefore consistent with the many reports of impaired sleep and SDB across neurodevelopmental disorders. Nevertheless, subtle differences are present. Individuals with Rett syndrome for instance, often exhibit sleep apnea hyperventilation and breath-holding spells, which can lead to significant disruptions in sleep architecture [46]. On the other hand, children with Prader-Willi syndrome and Fragile X frequently often experience obstructive sleep apnea, a contributing factor to increased daytime sleepiness [47, 49]. These parallels underscore the importance of monitoring and managing respiratory function in neurodevelopmental disorders to improve overall health outcomes and quality of life.

Also children with epilepsy exhibit a higher prevalence of sleep disordered breathing compared to their healthy peers [50]. Major risk factors for sleep apneas and sleep-disordered breathing in pediatric epilepsy patients include poor seizure control and antiepileptic drug polytherapy [50]. For example, recent nonclinical data show that combining GABA_A_ positive allosteric modulators and opioids can lead to respiratory depression in rats [51]. Also seizures themselves have been shown to negatively impact respiration [52–54]. Conversely, respiratory problems and reduced oxygen saturation during sleep may worsen epilepsy. The use of benzodiazepines, which is more common in cases of severe epilepsy, may further impact respiratory function [55, 56]. While the respiratory depressant effects of benzodiazepines and barbiturates are well-known [28, 57–61], the respiratory impact of many other co-medications observed in this study remains unclear. Some medications could potentially contribute to weight gain, which, when combined with hyperphagia—a symptom of AS that can lead to obesity—might further exacerbate respiratory difficulties [62]. This is of particular concern given the link between seizure-induced respiratory depression and sudden unexpected death in epilepsy (SUDEP) [52–54]. The cause of reduced RR observed in AS is therefore difficult to pinpoint and is likely the result of a complex interplay of factors, including genetic vulnerability, neurotransmitter dysregulation, epilepsy, and effects of medications [63–65]. Our findings suggest further research on respiratory abnormalities in AS.

Analysis of age-corrected Bayley scores within the deletion AS subgroup suggests that a more pronounced bradypnea phenotype is accompanied by higher symptom severity, in particular with poorer performance in expressive and receptive communication. It is not possible to infer any causal relationship from this correlative observation but it is a possibility that bradypnea-related chronic hypoxemia in the low respiration rate group be a contributing factor to the increased symptom severity. This is particularly concerning given the already compromised neurodevelopmental status of these individuals. Moreover, the lower oxygen saturation levels observed, may indicate an increased risk for sleep-related breathing disorders not limited to bradypnea, but could extend to OSA, a well known risk factor for cardiovascular complications, metabolic disturbances and impaired cognitive and intellectual functioning [66]. In patients with ADHD, the presence of SDB, including OSA, has been implicated in exacerbating ADHD symptomatology [67] and interventions targeting OSA have been reported to ameliorate cognitive symptoms in these patients [68]. Despite deliberately excluding periods indicative of potential sleep apnea from our analysis, these findings support exploration of targeted interventions to enhance nocturnal respiration in deletion AS. Such interventions, including the careful management of seizures without impairing respiratory function, could potentially improve both sleep quality and cognitive outcomes for individuals with AS. Moreover, a case study involving a pediatric patient with AS who suffered from central hypoventilation showed that respiratory rates during sleep improved after the patient’s medication was switched from clobazam, a GABA_A_ receptor positive allosteric modulator contraindicated for those with respiratory issues, to levetiracetam, which does not influence GABAergic transmission [16]. The observed higher frequency of benzodiazepine use among the low respiratory rate group in our study raises important questions about the interplay between medication effects and the severity of AS symptoms, particularly in deletion carriers. While it is challenging to disentangle the observed respiratory effects from the inherently more severe phenotype of AS in deletion carriers (see also last paragraph), our findings raise some questions regarding Benzodiazepine use and highlights the urgent need for further research to unravel the intertwined genetic and pharmacological factors contributing to respiratory depression in AS.

Despite our efforts to minimize the impact of body movements and seizure activity on the respiratory data through preprocessing, the potential influence of interictal epileptiform discharges (IEDs) and tonic seizure activity, a common feature of AS, on the BCG signal cannot be entirely excluded. Additionally, the exclusion of segments with large body movements or clonic seizures, while necessary for data integrity, may have resulted in the omission of relevant respiratory events. Therefore, our findings should be interpreted with caution, and further research is needed to fully understand the interplay between seizure activity and respiratory patterns in AS.

## Conclusion

This study describes a bradypnea phenotype in individuals with deletion AS that goes in hand with reduced blood oxygen level. Our correlative findings do not allow disentangling the causal relationships between various factors including medication, genetics and related changes in brain function, epilepsy and sleep, but do suggest further investigation of the bradypnea phenotype in AS and possibly other neurodevelopmental disorders. Furthermore, our work suggests ballistocardiography implemented in a sleep mat as a valid tool for long-term, low-burden monitoring of respiration, that is well suited for children with intellectual disability.

## Electronic supplementary material

Below is the link to the electronic supplementary material.

Supplementary Material 1

## Data Availability

The datasets used and/or analyzed during the current study are available from the corresponding author on reasonable request.
