# Supplementary material for: Lower respiratory rate during sleep in children with Angelman syndrome compared to age-matched controls
Source: Orphanet J Rare Dis. 2025 Apr 8;20:167. doi: 10.1186/s13023-025-03553-2 (PMC11980168; doi:10.1186/s13023-025-03553-2)
Supplement: Supplementary file 1 — Supplementary Material 1 [file 13023_2025_3553_MOESM1_ESM.docx]

**Supplementary material**

**Supplementary Methods 1:**

**Signal processing workflows and quality control of BCG data**

The sleep mat is programmed to start recording as soon as someone enters the bed and stops recording after 20 minutes without bed presence. After an additional delay the device starts to securely transmit the recorded data to a remote server at Emfit. If the device is cut from power directly after leaving the bed this process is interrupted and leads to disrupted data. The resulting interrupted/damaged data segments often span more than 24h as no proper stop signal could be registered and have to be excluded. Additionally in some cases data segments were recorded during the day if participants spent time in bed for naps or other activities. To focus solely on nightly sleep only we excluded data recorded during the day. Additionally multiple data segments per night are written if a participant leaves their bed for more than 20 minutes in such cases the segments are merged to receive one record per night and participant. This is done by combining the arrays of 4s bins of all segments from the same night and by building new stats.

The resulting dataset comprises 18’118 data segments from 72 study participants that underwent further quality control. Here we focus on the subset of 60 pediatric participants (see Methods) whose data comprise 14’866 data segments. In a first step all data segments that could not be linked to nightly sleep have been excluded based on the following rules:

The segment must start between 6pm-6am OR cross midnight, while NOT crossing 3pm (less than 24h). If multiple valid data-segments per night are recorded, segments are merged to a single night.

A total of 2’936 data-segments underwent merging. After this basic QC and merging the dataset comprised 12’629 valid data-segments, i.e. nights of data.

Respiration specific QC bcg-data:

Overall “time in bed” ~125’912 hours of data; average time in bed 9.97h/night;

While activity data is always registered, heavy movement can confound respiration and heart rate data and the device is set to write respiration/heart rate data only if the signal is of sufficient quality to extract these data. For the ~125’912 hours of activity data ~84’409 hours (67% coverage) of respiration data have been registered by the devices.

This results in a total number of 75’968’306 sleep mat 4s bins of respiration data.

To exclude data with potential apnea events or other artifacts we filtered the remaining values of respiration rate with a minimum of 8 breaths per minute (bpm). This value was used to also include data with unusual low values for this age group. The upper frequency of the respiration rate data is limited by the firmware of the devices (will be changed in future versions) to 25.5 bpm. Resulting in all values above 25.5 set to 25.5. This led to the decision to only include values below 25.5 bpm. This filter step resulted in 66’803’381 4s bins between 8 and 25.5 bpm.

In a last step we excluded all nights with less than 3 hours of respiratory data to be sure to have only nights spanning a substantial fraction of sleep.

This resulted in a dataset comprising 10’040 nights 63’299’299 4s rr_bins ~70’332 hours of valid respiration data (Nights per participant: mean=139.44, median=122, range: 4-436, SD = 99.59; ~7 hours of respiration data per night).

**Supplementary Methods Figure 1. Outline of BCG data preprocessing workflow**


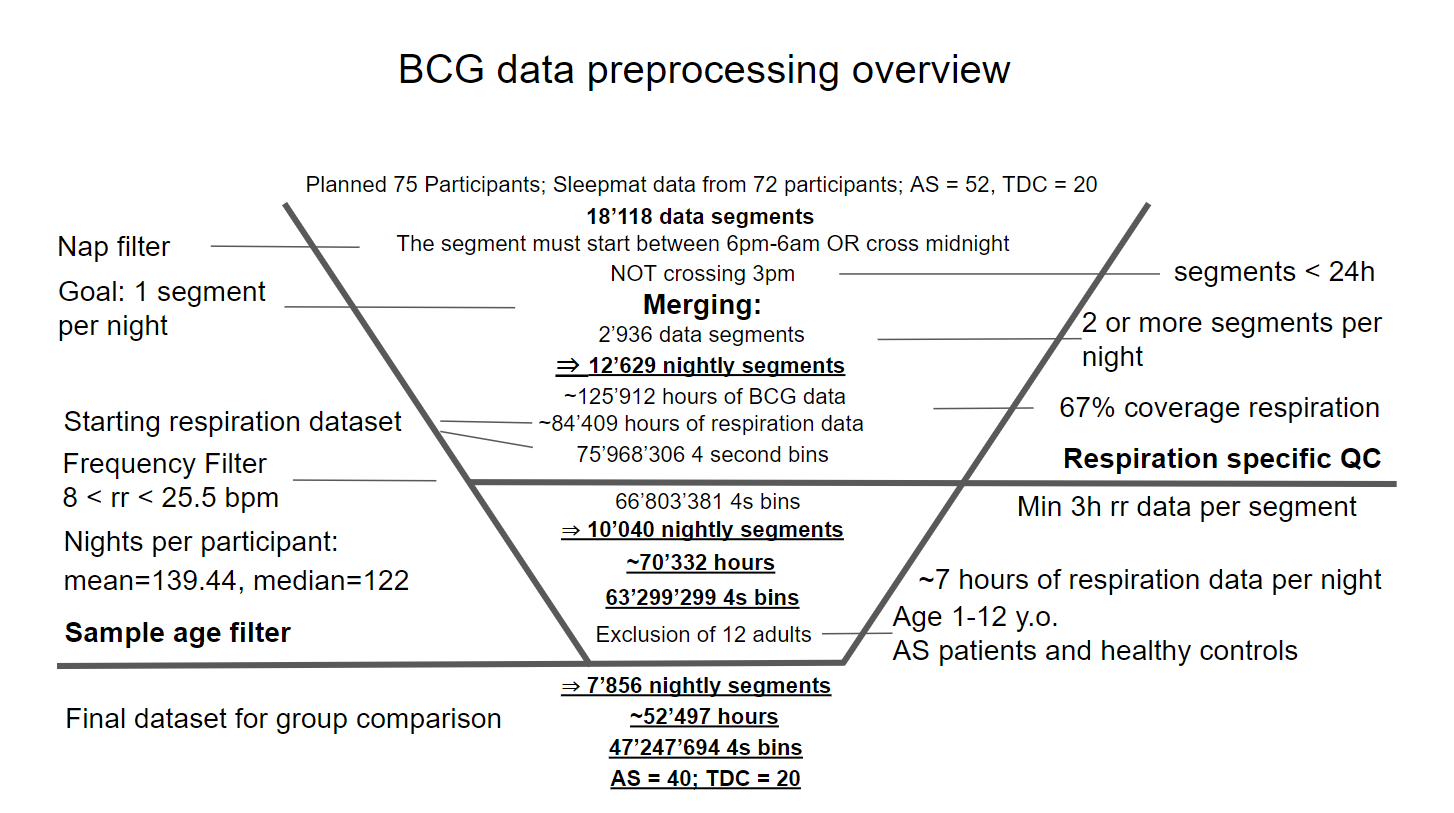


**Supplementary Methods 2:**

**Signal processing workflows and quality control of PSG data**

Overnight polysomnography (PSG) was performed in the participants’ homes by Neurotech (Neurotech, Waukesha, WI, USA). Data were recorded with Trackit T32/T4A recorders with Fasttrack effort interface kits with abdominal belts for respiration and XPOD Pulse Oximeter for SpO2 levels at 400 Hz and saved in the european data format (edf).

Further information on home visit PSG data collection can be found in the FREESIAs landmark publication [[1]](https://app.readcube.com/library/5579a63f-eba2-4aa9-93d2-c07f0f711532/all?uuid=30411082367656506&item_ids=5579a63f-eba2-4aa9-93d2-c07f0f711532:c7b23c43-b676-495a-8512-22bafa9cb891).

PSG preprocessing: Raw signal data were extracted from edf files using “edfrd v0.7” python library using start and stop time points defined by EEG sleep staging starttime first sleep and endtime last sleep.

Sleep staging based on PSG is challenging in individuals with Angelman syndrome due to the abnormally high EEG delta power phenotype [[2]](https://app.readcube.com/library/7e6be62c-367e-412b-a797-b60ccddc36e3/all?uuid=23066460341396233&item_ids=7e6be62c-367e-412b-a797-b60ccddc36e3:3297af49-9ebf-4243-9d3f-e70e35a7975c). To ensure that included respiration data originates from sleep, we used a conservative approach to define definite sleep episodes based on the overnight EEG spectrogram. For this approach, overnight spectrograms were visually inspected. Sleep was defined as periods of increased delta power that were distinctly different from definite wake periods confirmed during periods with lights on before subjects fell asleep.

The data from all channels were loaded and the respiration belt and pulse oximetry signal was extracted for further processing. For respiration belt data processing two parallel approaches were applied one for the extraction of respiratory rate the second to extract data quality across time.

**Signal processing workflows and quality control of PSG respiration belt data**

1. Peak based approach (respiratory rate)

The neurokit v0.2.1 respiration pipeline (rsp_clean, rsp_peaks, rsp_rate) was used to extract respiration rate. In a second step the data were resampled to match 32s bin resolution using the neurokit signal_resample function with method “interpolation” to be fused with the qc dataset received by the second approach. Beside a relatively simple data cleaning consisting of linear detrending followed by a fifth order low-pass IIR Butterworth filter [[3]](https://app.readcube.com/library/7e6be62c-367e-412b-a797-b60ccddc36e3/all?uuid=3102434672446265&item_ids=7e6be62c-367e-412b-a797-b60ccddc36e3:40fa67c4-f0c5-4f41-b2e5-22cb542f1ced) the pipeline doesn’t provide detailed data-quality information across time. As the collected data are of mixed quality, containing segments where the signal is disturbed e.g. by body movements, we applied a parallel processing approach to get dense quality information across time.

1. FFT approach (QC information)

Matplotlib.pyplot.specgram was used to calculate a spectrogram with a 64 seconds window size with 32 seconds overlap. Frequencies were prefiltered within frequencies 0-0.5Hz and the max power values within this frequency band were selected as the dominant frequency for respiration rate per window resulting in a 32 sec resolution dataset. In a second step a sliding window approach of 20 * 32 seconds was applied to extract variance of the max-power values.

During inspection of the respiration belt data we detected data segments where the respiration belt probably shifted position or the patient moved and the signal was lost. For those segments no clear max frequency could be extracted within the predefined frequency range (max spectral power values randomly distributed) and as a result the variance of the max frequency values per window went up. Based on this finding we used variance as an additional qc for the detection of data segments with bad data quality per 20 * 32 sec window.

The merged data (neurokit respiratory rate & variance of max power values) were filtered for the frequency of the respiration rate from neurokit (8/60-0.425Hz; 8-25.5 bpm) and variance from the FFT approach (var < 0.01Hz) per 20 * 32 second window. The combination of frequency and variance filters were applied on the one hand to exclude bad quality segments (variance) and on the other hand to match the frequency spectrum of the ballistocardiography device for direct comparison. After basic QC, records with less than 3h of good quality data were excluded.

**Signal processing workflows and quality control of blood oximetry** **data**

For a subset of the PSG recordings, SpO2 measures were available in the edf files.

After extraction the SpO2 signal underwent mean resampling with a 32 second window size (to get the same time resolution/axis like respiration belt data). Variance and median was calculated for 20 * 32 second (~10 min) sliding windows. Data segments with variance > 50 were excluded as high variance represents physiologically implausible signal changes indicating potential temporary signal loss of the finger sensor due to movements. Additionally values >= 100 were excluded as SpO2 levels max at 100% and empty values are at 127. After basic QC records with less than 3h of high quality data were excluded. The resulting dataset comprised 19 nights for AS 1-12 N=9 (avg age = 5.77), TDC 1-12 N=7 (avg age = 4.71) whereas one AS patient and two healthy controls had 2 nights of data. For the three participants the average across the two measures was built for further analysis.

**Supplementary methods Figure 2. Outline of PSG data preprocessing workflow**


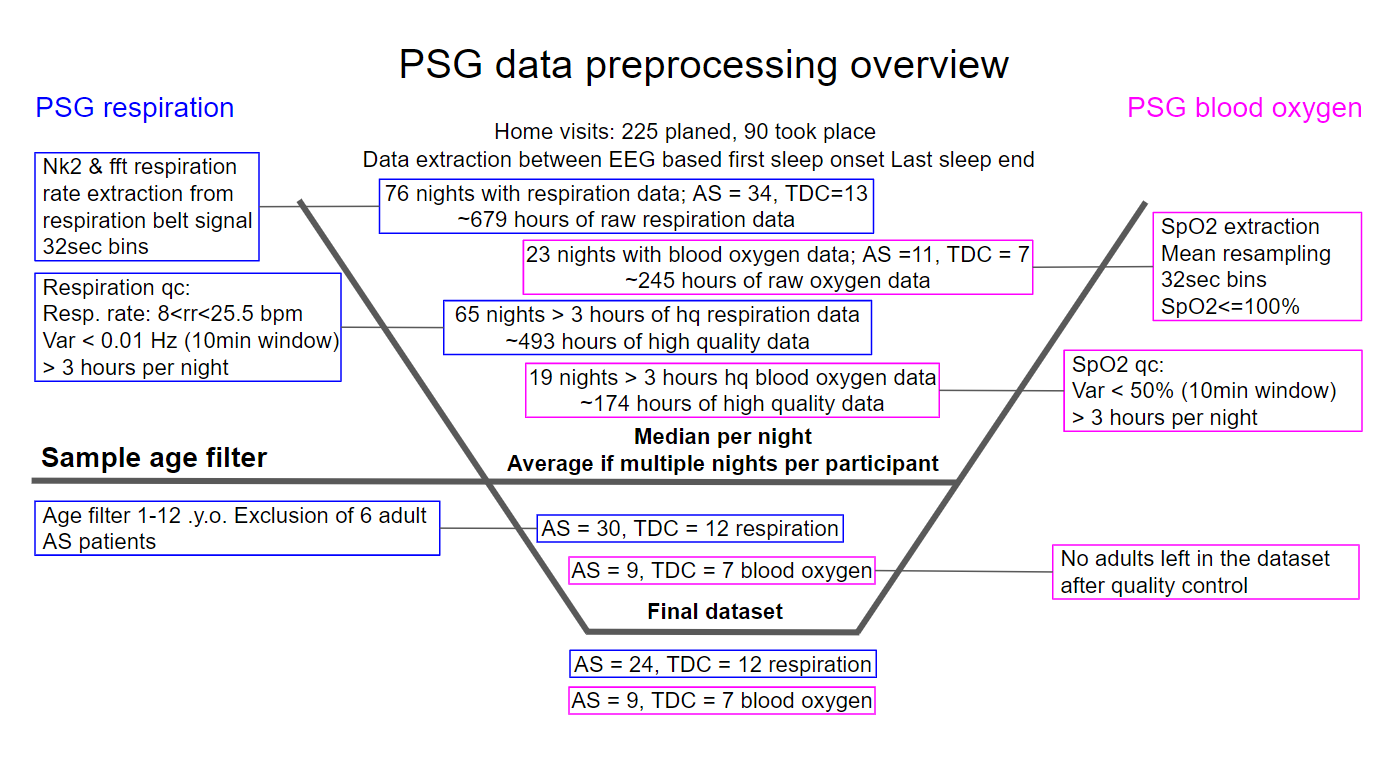


**Supplementary results**

The medications were extracted for our target population of AS patients 1-12 y.o. N = 40

**Supplementary Results Table 1**

| Ongoing medication | | | Daily medications | | |
| --- | --- | --- | --- | --- | --- |
| **compound** | **Number of subjects** | **Relative count** | **compound** | **Number of subjects** | **Relative count** |
| melatonin | 18 | 45 % | clobazam | 14 | 35 % |
| clobazam | 14 | 35 % | levetiracetam | 14 | 35 % |
| levetiracetam | 14 | 35 % | melatonin | 13 | 32.5 % |
| CBD* | 11 | 27.5 % | CBD* | 11 | 27.5 % |
| clonazepam | 10 | 25 % | clonazepam | 7 | 17.5 % |
| diazepam | 10 | 25 % | clonidine | 6 | 15 % |
| clonidine | 6 | 15 % | trazodone | 4 | 10 % |
| cetirizine | 4 | 10 % | diphenhydramine | 3 | 7.5 % |
| diphenhydramine | 4 | 10 % |  |  |  |
| ibuprofen | 4 | 10 % |  |  |  |
| trazodone | 4 | 10 % |  |  |  |
| albuterol | 3 | 7.5 % |  |  |  |
| paracetamol | 3 | 7.5 % |  |  |  |

AS patients suffer from a complex mix of symptoms that need to be treated. Seizures, sleep and digestion problems are normally treated with medications additionally nutritional supplements are often applied which results in a multitude of potential drug interactions. For simplicity we excluded food supplements and created a simplified overview about the most common drugs that might have an impact on respiratory rate (used by min 7.5% of the patients). The difference between the two tables allows us to estimate which fraction of drugs are taken daily or just here and then. E.g. For melatonin the drop from 45% to 32.5% between Ongoing and Daily indicates that 12.5% of the participants do not get melatonin daily. CBD*: cannabidiol containing products.

E.g. 6 y.o. Boy: “levetiracetam, lorazepam, CBD oil, melatonin, diazepam, ondansetron”

**Supplementary Results Figure 1**


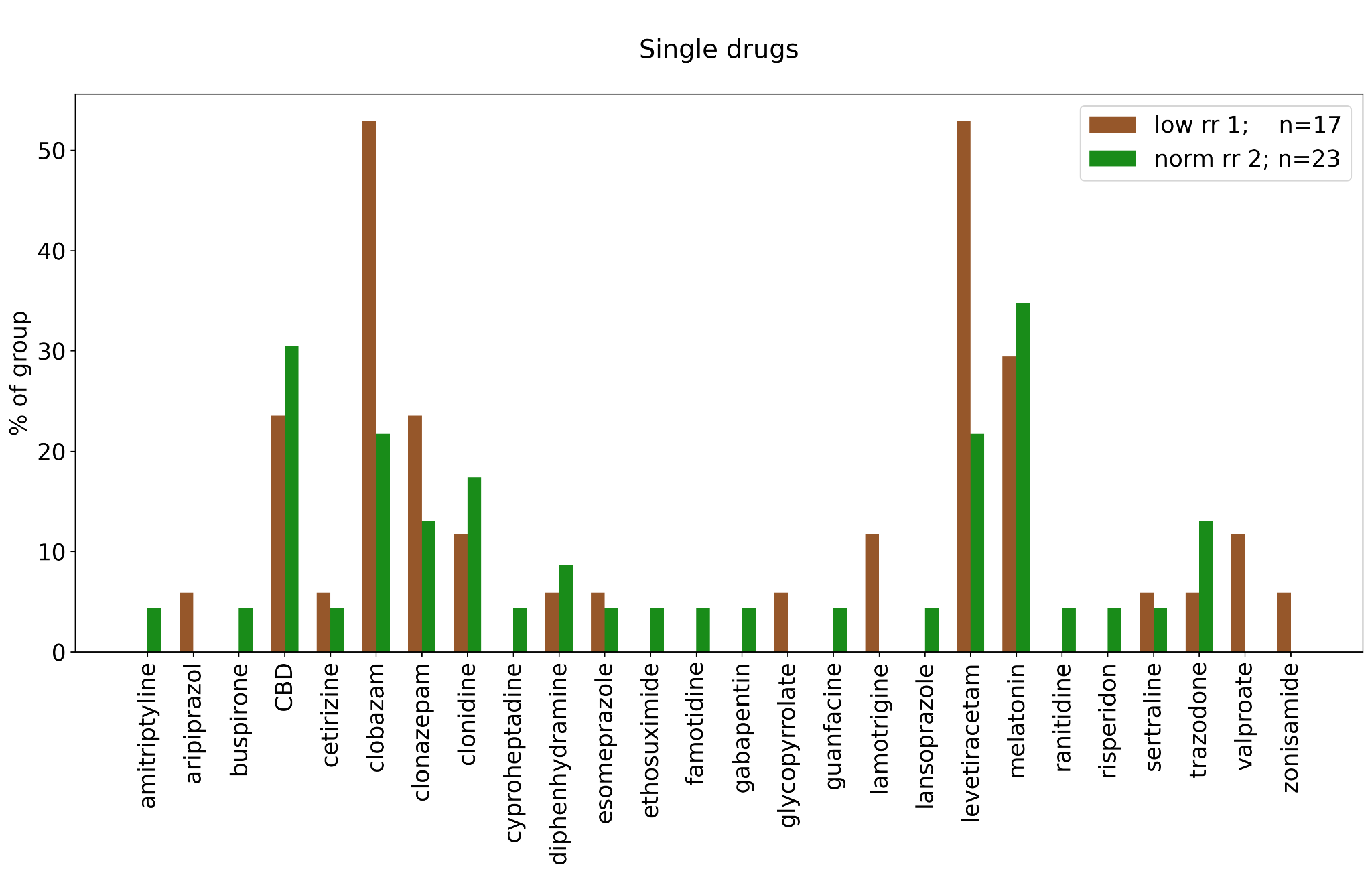


This figure presents the distribution of individual medications daily used by AS individuals categorized by respiratory rate: lowRR (< 13.8 breaths/min) and normRR (> 13.8 breaths/min). Medications are listed individually and are grouped according to deletion and non-deletion carrier status. The figure provides a detailed view of the specific medications taken by patients across respiratory rate categories.

**Supplementary results Figure 2: Age Angelman vs Controls**

**
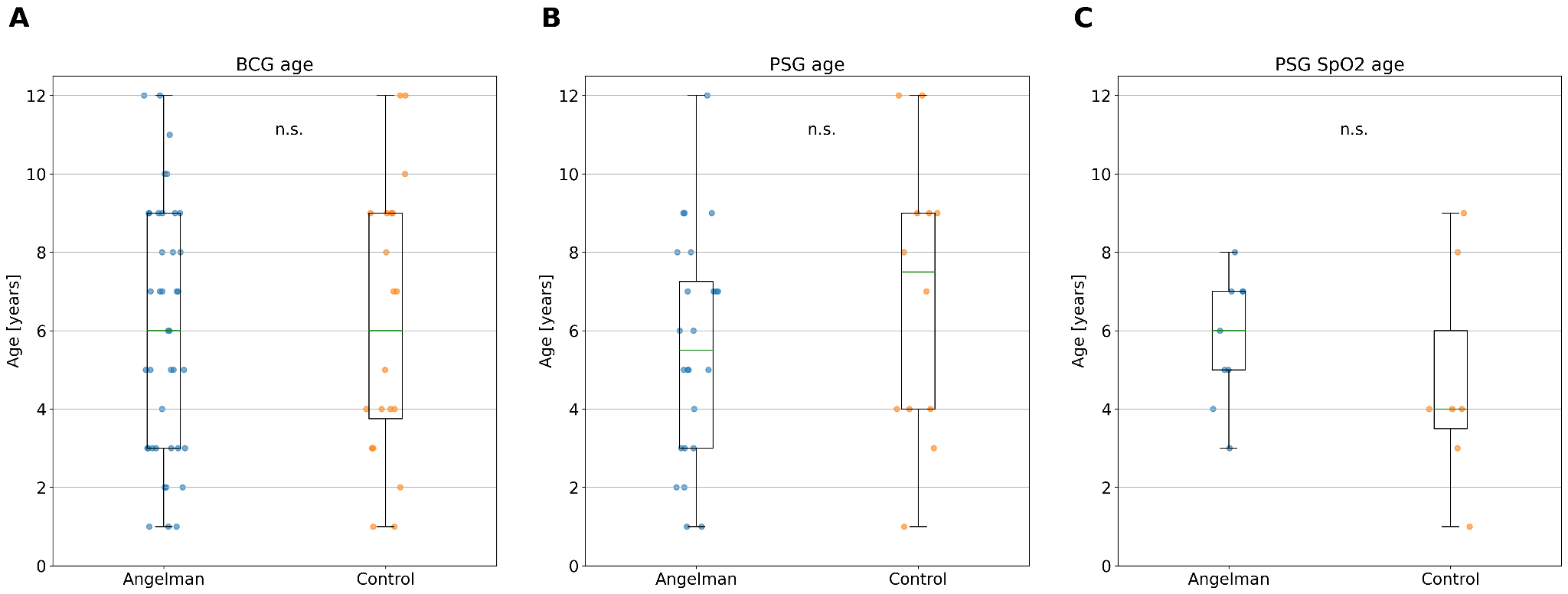
**

**Fig. Age:** Age as a proxy for height and weight in children has been reported to have a direct connection to sleep respiratory rate. This connection has been reported to be nonlinear and our sample size for healthy controls is not sufficient to elaborate normative data. Generally older age is associated with lower respiratory rate.

**A Age BCG respiration** AS 1-12: N=40, age(median) = 6 y.o.; TDC 1-12: N=20, age(median) = 6 y.o. (Mann Whitney: Stats = 386.5, p-value = 0.837; BMT = 0.205, p-value = 0.838) **B Age PSG respiration belt** data after qc AS 1-12: N=24, age(median) = 5.5 y.o. TDC 1-12: N=12, age(median) = 7.5 y.o. Mann Whitney: Stats = 112.5; p-value = 0.295 BMT = 0.994; p-value = 0.335 No significant age differences in the PSG dataset; Non significant age difference between AS and TDC potentially weakens respiratory effect a little bit. **C Age PSG SpO2** data set after qc AS 1-12 N=9 age(median) = 6; TDC 1-12 N=7 age(median) = 4, BMT = -0.833, p-value=0.430

**Age BCG genetic groups**

**Supplementary Results Figure 3**

AS deletion: median age = 5 y.o.; Non-deletion = 7 y.o.; TDC = 6 y.o.


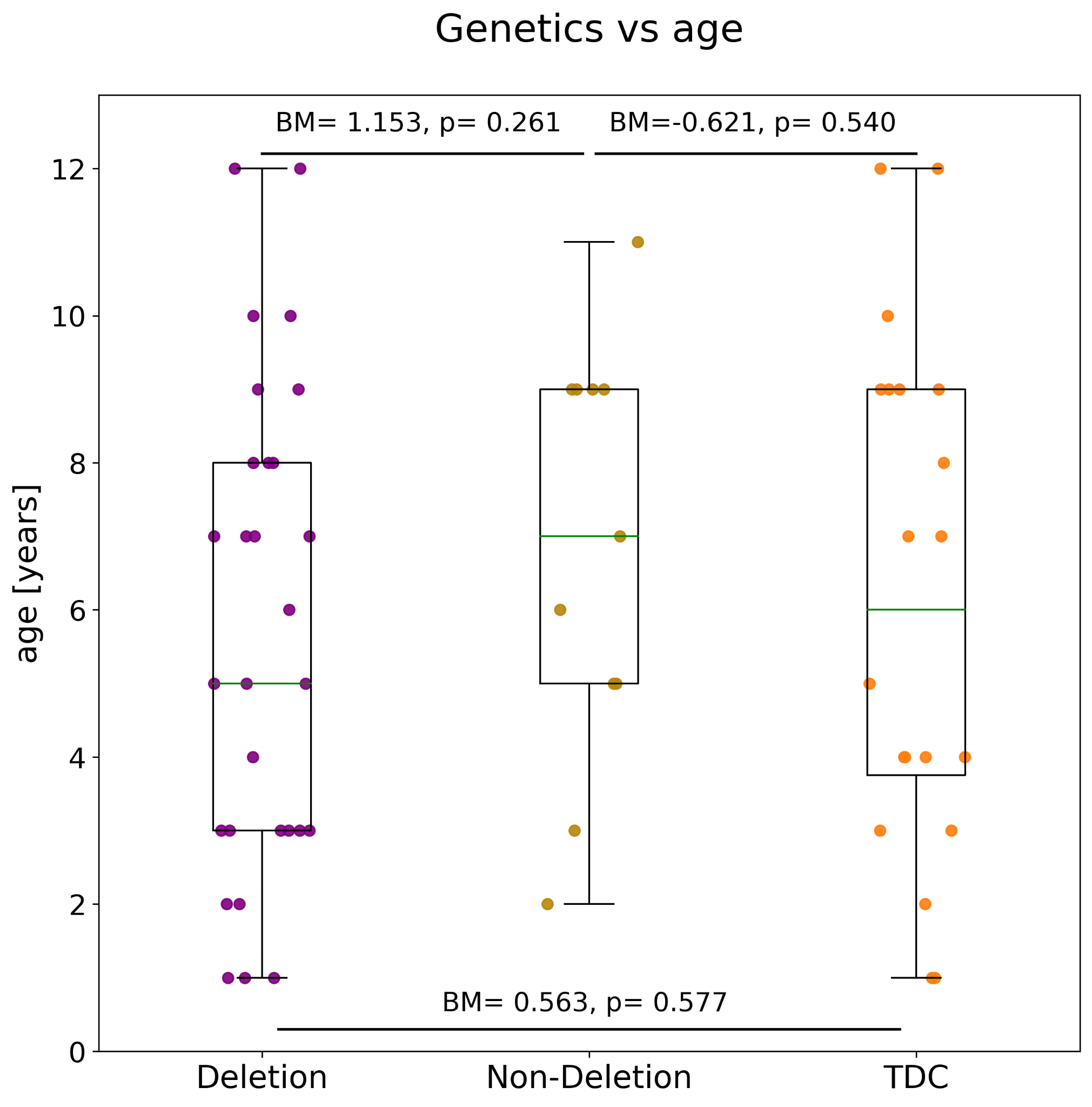


**Bibliography supplements**

[1. Tjeertes J, Bacino CA, Bichell TJ, Bird LM, Bustamante M, Crean R, et al. Enabling endpoint development for interventional clinical trials in individuals with Angelman syndrome: a prospective, longitudinal, observational clinical study (FREESIAS). J Neurodev Disord. 2023;15:22.
2. Frohlich J, Bird LM, Dell’Italia J, Johnson MA, Hipp JF, Monti MM. High-voltage, diffuse delta rhythms coincide with wakeful consciousness and complexity in Angelman syndrome. Neurosci Conscious. 2020;2020:niaa021-.
3. Khodadad D, Nordebo S, Müller B, Waldmann A, Yerworth R, Becher T, et al. Optimized breath detection algorithm in electrical impedance tomography. Physiol Meas. 2018;39:094001.](https://app.readcube.com/library/?style=Orphanet%20Journal%20of%20Rare%20Diseases+%7B%22language%22:%22en-US%22%7D)
